# Case Report: Deletion in the 5' untranslated region of *TAFAZZIN* in a boy with Barth syndrome

**DOI:** 10.3389/fcvm.2026.1766067

**Published:** 2026-02-16

**Authors:** Emma S. Singer, Janine Smith, Richard Lin, Ansley M. Morrish, Sean Lal, Claire Irving, Charlene Casey, Ingrid King, Robert G. Weintraub, Richard D. Bagnall

**Affiliations:** 1 Bioinformatics and Molecular Genetics Group at Centenary Institute, The University of Sydney, Sydney, NSW, Australia; 2 Faculty of Medicine and Health, The University of Sydney, Sydney, NSW, Australia; 3 Department of Paediatrics, The Children’s Hospital at Westmead, Sydney, NSW, Australia; 4 Department of Clinical Genetics, The Children’s Hospital at Westmead, Sydney, NSW, Australia; 5 Heart Centre for Children, The Children’s Hospital at Westmead, Sydney, NSW, Australia; 6 Murdoch Children’s Research Institute, University of Melbourne, Melbourne, VIC, Australia; 7 University of Melbourne, Melbourne, VIC, Australia; 8 Department of Cardiology, Royal Children’s Hospital, Melbourne, VIC, Australia

**Keywords:** 5' untranslated region, Barth syndrome, cardiomyopathy, start loss, *TAFAZZIN*

## Abstract

**Background:**

Barth syndrome is an X-linked disorder characterised by cardiomyopathy, growth abnormalities, neutropenia, and 3-methylglutaconic aciduria. It is caused by pathogenic variants in *TAFAZZIN*, which encodes a mitochondrial protein essential for cardiolipin remodelling. In this study, we describe the case of a patient with Barth syndrome in whom initial research genetic testing missed a 5' untranslated region deletion in *TAFAZZIN* that was later identified through a phenotype-guided reanalysis of exome sequencing data.

**Case presentation:**

A male infant presented with dilated cardiomyopathy at 7 months of age and underwent cardiac transplantation at 19 months. Initial comprehensive cardiac genetic testing was indeterminate. Subsequent clinical investigations recorded a slight increase in the levels of 3-methylglutaconic acid and intermittent neutropenia, and a history of intermittent neutropenia was noted in his mother and maternal grandmother, prompting a consideration of Barth syndrome. A reanalysis of exome sequencing data identified a hemizygous 116 base pair deletion spanning the 5' untranslated region and start codon of *TAFAZZIN*. An RNA analysis from the proband's cardiac tissue amplified truncated *TAFAZZIN* transcripts, and Western blotting confirmed the complete loss of full-length protein, consistent with the loss of the start codon and failure of translation initiation from a downstream in-frame methionine.

**Conclusion:**

We report a novel 116 bp *TAFAZZIN* deletion that prevents protein expression due to the loss of the canonical start codon. This case highlights the importance of including non-coding regions in genetic analysis and the diagnostic value of phenotype-guided reanalysis of genetic test data.

## Introduction

Barth syndrome (OMIM #302060) is a rare, life-threatening, X-linked recessive disorder characterised by cardiomyopathy, growth abnormalities, neutropenia, and elevated levels of 3-methylglutaconic acid (1). Affected individuals typically present with cardiomyopathy within the first year of life, often leading to heart failure and requiring heart transplantation (2). A delay from initial presentation to diagnosis of Barth syndrome is not uncommon (3). Symptoms are typically managed with diuretics, antiarrhythmics, and beta blockers (3).

Loss-of-function (LoF) variants in the *TAFAZZIN* gene, leading to reduced or absent enzymatic activity of the encoded TAFAZZIN protein, are the primary cause of Barth syndrome (4). *TAFAZZIN* is located on chromosome Xq28, with a full-length transcript comprised of 11 exons (*G4.5*, NM_000116.5). TAFAZZIN is a transacylase responsible for the remodelling of monolysocardiolipin (MLCL) to cardiolipin (CL) in the mitochondria (5). Cardiolipin is a phospholipid that is crucial for maintaining mitochondrial homeostasis by providing structural integrity to the inner mitochondrial membrane and supporting respiratory chain complex function during cellular respiration (6, 7). Disruption to the remodelling of CL leads to the accumulation of MLCL (8, 9). Therefore, clinically suspected Barth syndrome is typically confirmed through genetic testing for variants in *TAFAZZIN* and a biochemical analysis of the MLCL:CL ratio (10). Defective TAFAZZIN function and reduced CL levels alter the mitochondrial structure and hinder respiratory chain complex formation and function, although how this influences disease progression is relatively unknown (6, 7, 11).

In this study, we report the case of an infant with dilated cardiomyopathy (DCM) who underwent an indeterminate comprehensive cardiac genetic test, including *TAFAZZIN*. Subsequent development of neutropenia and 3-methylglutaconic aciduria prompted a reanalysis of genetic test data, which identified a 116 bp deletion from the 5' untranslated region (UTR) to the start codon of *TAFAZZIN.* RNA and protein analyses using explanted heart tissue from the proband confirmed the expression of truncated *TAFAZZIN* mRNA and the absence of full-length TAFAZZIN protein. Our case highlights the importance of including non-coding gene regions in genetic testing and the value of phenotype-guided genetic analysis.

## Patient and methods

The proband was recruited from The Children's Hospital at Westmead, Sydney, Australia. The parents provided written informed consent for a genetic testing (HREC Project Number 32092) and functional evaluation of heart tissue (HREC Project Number 38192), which were carried out in accordance with the ethics protocols approved by the Royal Children's Hospital Melbourne Human Research Ethics Committee. The parents provided written consent for the publication of a clinical case report.

### Cardiac genetic testing

Exome sequencing was performed on DNA extracted from fresh peripheral blood of the proband using previously published methods (12). Analysis was limited to rare variants in 202 genes associated with childhood-onset cardiomyopathy, as previously described in detail (12). A manual inspection of *TAFAZZIN* sequencing reads was performed using the Integrative Genomics Viewer, v.2.19.1 (13).

### Segregation analysis

DNA was extracted from fresh peripheral blood of the proband's parents, as previously described (12). Polymerase chain reaction (PCR) amplification across the *TAFAZZIN* deletion interval was performed using the GoTaq® Flexi DNA polymerase kit (Promega, Wisconsin, USA), with 40 ng DNA, 8% dimethyl sulfoxide (DMSO), and the following primer pair that was annealed at 60 °C for 35 PCR cycles: forward 5'-CTCCCCAGTGACGAGAGAGC and reverse 5'-GGTCCAGAAGCAGCTGTAGG. PCR products were resolved on an agarose gel.

### Human heart tissue

Left ventricular tissue of the proband, immediately snap-frozen in liquid nitrogen at surgical removal, was obtained from The Melbourne Tissue Heart Bank, Melbourne, Australia (HREC 38192). Control myectomy samples from adults aged between 21 and 55 years at the time of sample collection were obtained from the Sydney Heart Bank (14).

### RNA extraction and reverse transcription-PCR amplification

RNA was extracted from 20 µg snap-frozen myectomy tissue using 1 mL of TRIzol™ reagent (Life Technologies, California, USA), reverse-transcribed using random hexamers (Thermo Fisher, Massachusetts, USA), and PCR-amplified as previously published (15). The following are the PCR primers that were annealed in the 5' UTR and exon 3 of *TAFAZZIN* (NM_000116.5): forward 5'-CTCCCCAGTGACGAGAGAGC and reverse 5'-ACGCATCAACTTCAGGTTCC. PCR products were purified using Exonuclease I and Antarctic Phosphatase (New England Biolabs, Massachusetts, USA), resolved on an agarose gel resolved, and extracted using the NucleoSpin® Gel and PCR Clean-up kit (Macherey-Nagel, Düren, Germany). Sanger sequencing was performed at Macrogen, Inc. (Seoul, South Korea) and electropherograms were analysed using Sequencher v.5.4.6 (Gene Codes, Michigan, USA).

### Protein extraction and Western blotting

Fifty micrograms of snap-frozen left ventricular tissue was homogenised in the Pierce™ RIPA Buffer (Thermo Fisher) containing PhosSTOP™ (Roche, Basel, Switzerland) and cOmplete™, Mini, EDTA-free Protease Inhibitor Cocktail (Roche), using a Polytron PT 2100 homogeniser (Kinematica, Lucerne, Switzerland). Protein extraction and Western blot analysis was performed as previously published (16). The Western membrane was stained post-transfer with Ponceau S Red (Sigma-Aldrich) and horizontally cut at approximately 50 kDa, relative to the PageRuler™ Plus Prestained Protein Ladder loaded onto a NuPAGE™ 4%–12% Bis-Tris Protein Gels, 1.5 mm, 15-well (Thermo Fisher), with the 1X 3-morpholinopropane-1-sulfonic acid (MOPS) running buffer, prior to blocking in 5% skim milk powder in phosphate buffered saline with tween (PBS-T). The anti-MYBPC3 monoclonal antibody (1:1,000, Abcam, ab108522) was hybridised in the upper half of the membrane and the anti-TAFAZZIN monoclonal antibody (1:1,000, Abcam, ab307148) was hybridised in the lower half of the membrane in 5% BSA in PBS-T. The goat anti-rabbit horseradish peroxidase (HRP) conjugate (1:10,000, Invitrogen, G21234) secondary antibody was hybridised in 5% skim milk powder in PBS-T.

## Results

**Figure 1:**
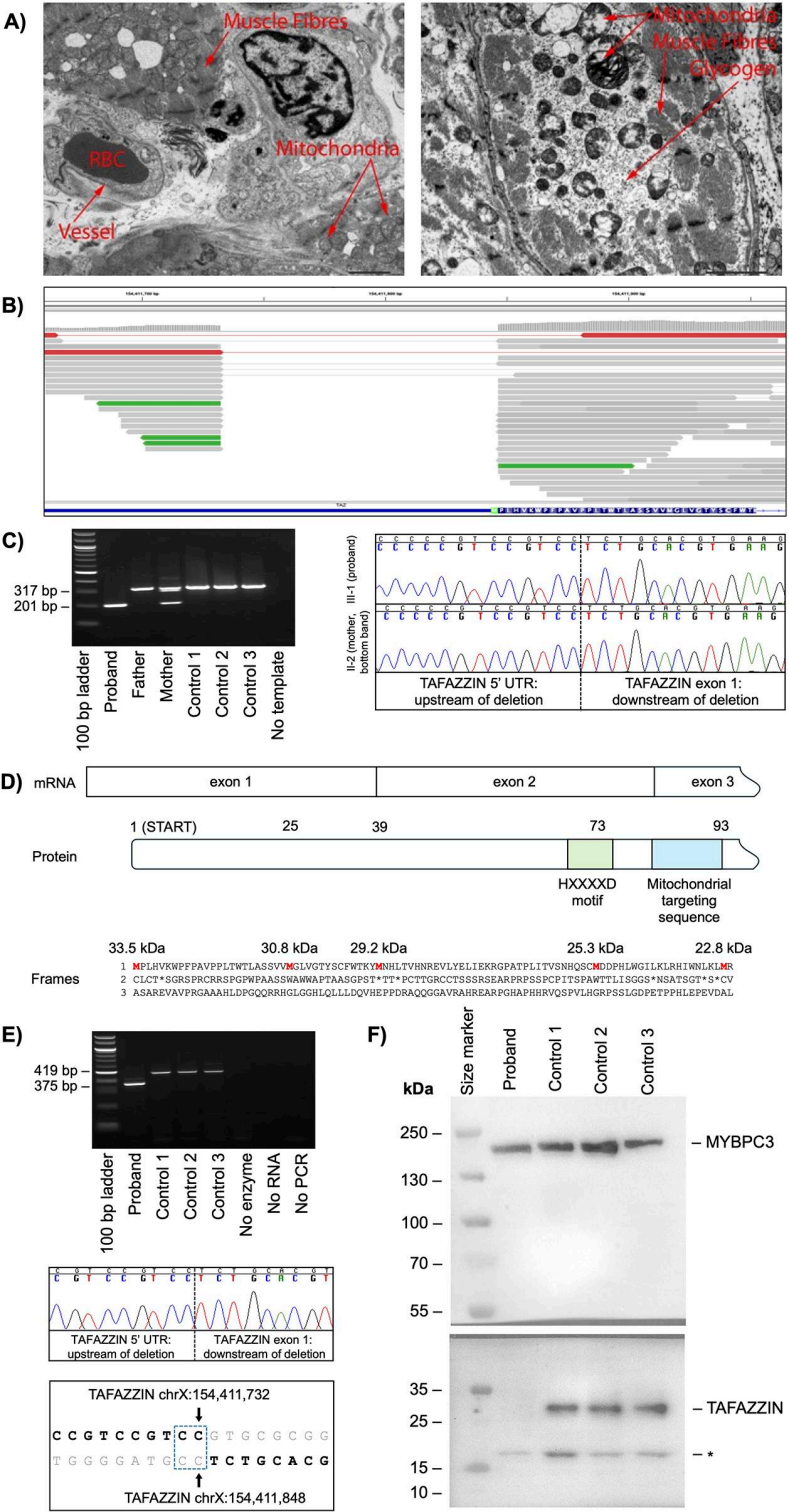
5' untranslated region deletion in *TAFAZZIN* causes the loss of full-length TAFAZZIN. **(A)** Electron microscopy of explanted heart tissue of the proband. Images show no obvious increase in the number of mitochondria or a conspicuous irregularity in the arrangement of cristae. Glycogen particles are normal in amount and appearance. Contractile elements of the myocytes show no obvious abnormality. RBC, red blood cell; scale bar, 2.0 μm. **(B)** Exome sequencing alignments mapped to *TAFAZZIN* highlighting a deletion in the 5' untranslated region and start codon (green box). **(C)** PCR (left panel) and Sanger sequencing (right panel) confirming a maternally inherited 116 bp genomic deletion in *TAFAZZIN*. **(D)** A schematic showing the beginning of the TAFAZZIN mRNA exon structure (top), with the amino acid number of all AUGs indicated (middle) and the protein sequence across all three frames with the calculated molecular weights of proteins commencing at all theoretical methionine residues in red (bottom). **(E)** RT-PCR (upper panel) and Sanger sequencing (middle panel) confirmed the 116 bp deletion in *TAFAZZIN* transcripts, with the breakpoint junction sequences (bold sequence, lower panel) showing a region of microhomology (blue stippled box). **(F)** Western blot confirming the absence of TAFAZZIN in the proband, with MYBPC3 loading control. The PageRuler™ Plus Prestained Protein Ladder was used as the marker, with molecular weights shown in kilodaltons (kDa). The asterisk (*) indicates an unconfirmed protein band. Bp, base pair; UTR, untranslated region.

The male proband presented with cough, coryza, poor feeding, and increased work of breathing. A chest X-ray showed cardiomegaly and an echocardiogram showed a severely hypocontractile left ventricle with impaired systolic function and a dilated left atrium. He was diagnosed with DCM at 7 months of age and he developed progressive congestive heart failure (NYHA Class IV) with a left ventricular ejection fraction of 26%. He was initially managed with diuretics, a beta-blocker, angoitensin converting enzyme (ACE) inhibitor, and continuous positive airway pressure (CPAP) respiratory support but later received a ventricular assist device implant before undergoing a cardiac transplantation at the age of 19 months. A histology of myocardial tissue retrieved at the time of the ventricular assist device implant showed mild interstitial fibrosis, perivascular lymphocytes, plasma cells in the epicardium and subepicardial zones, and endocardial fibroelastosis, consistent with his clinical diagnosis of DCM. Electron microscopy of myocardial tissue did not show obvious myocytes or mitochondrial abnormalities (Figure 1A).

An exome-based analysis of a comprehensive 202 cardiac disease gene panel, including *TAFAZZIN*, did not identify a genetic cause of cardiomyopathy (12). The proband’s family was of European ancestry and there was no history of cardiomyopathy or Barth syndrome.

The proband subsequently developed occasional muscle fatigue and recorded slightly elevated levels of 3-methylglutaconic acid and intermittent neutropenia consistent with a diagnosis of Barth syndrome. This clinical diagnosis prompted a manual re-inspection of available exome sequencing data across *TAFAZZIN* and revealed a hemizygous 116 bp deletion of the 5' UTR and start codon; chrX(GRCh38):g.154411733-154411848del; NM_000116.5(*TAZ):*c.-111_5del (Figure 1B). This deletion was missed during initial genetic testing because only three base pairs fell on the edge of the coding regions that were the target of the deletion analysis. A segregation analysis revealed that the deletion was maternally inherited (Figure 1C).

The *TAFAZZIN* deletion had uncertain clinical significance due to its unknown impact on transcription of the gene, and an in-frame methionine 24 amino acid residues downstream of the start codon might initiate the translation of a slightly shorter, potentially functional protein (Figure 1D). To determine the impact of the deletion on transcription, RNA was extracted from the heart tissue of the proband and three control hearts. RT-PCR amplification of *TAFAZZIN* transcripts with a 116 bp deletion in the proband and full-length transcripts in the control hearts confirmed that the *TAFAZZIN* promoter was still intact and able to support transcription (Figure 1E).

To determine whether the translation of TAFAZZIN is initiated from an alternative start site, protein was isolated from the proband's explanted heart tissue and three myectomy control hearts. A Western blot analysis showed no full-length TAFAZZIN protein in the proband's heart tissue compared with controls (Figure 1F). One small protein band of approximately 17 kDa was observed in all samples, but this did not correspond to the mass of any known TAFAZZIN protein isoform. We concluded that the loss of the canonical translation start site led to the complete loss of full-length TAFAZZIN without any expression of a truncated isoform, i.e., a protein LoF consistent with Barth syndrome.

We classified the deletion using the 2015 American College of Medical Genetics and Genomics/Association for Molecular Pathology (ACMG/AMP) variant classification framework in combination with the PVS1 decision tree for LoF variants (17, 18). The deletion is absent in the gnomAD general population database (PM2); the phenotype is highly specific for TAFAZZIN-related Barth syndrome (PP4); the deletion results in a start loss and the altered region plays a critical role, as demonstrated by no detectable full-length TAFAZZIN protein (PVS1_strong), supporting a likely pathogenic classification that was confirmed by a clinical genomics test. A similar deletion that removes the first 36 amino acids of TAZAFFIN (NM_000116.3 c.−72_109+51del) was found in a boy with Barth syndrome (19). The deletion in our proband also overlaps the first intron of *DNASE1L1* (NM_001009932.3), encoded on the reverse DNA strand. The deletion classifies as a rare variant with respect to *DNASE1L1*, as the gene currently has no definitive role in a Mendelian disorder.

## Discussion

We report a novel, likely pathogenic, 116 bp maternally inherited deletion spanning the 5' UTR and start codon of *TAFAZZIN* in an infant with Barth syndrome. In a registry report of 73 patients with Barth syndrome, 48 (66%) presented with cardiomyopathy within the first year of life, yet the mean age at clinical diagnosis of Barth syndrome was 4.0 ± 5.5 years due to diagnostic delays (2). Our proband presented with DCM in infancy, progressing to heart failure requiring cardiac transplantation. Initial research genetic testing missed the deletion in *TAFAZZIN* that would have confirmed Barth syndrome. This was attributed to the deletion location in the upstream non-coding region of the gene, with only the ATG start codon part of intervals analysed for deletions. To avoid such false negatives, genetic testing intervals should include all transcribed gene regions, including upstream and downstream untranslated regions. A histology of explanted heart tissue did not identify characteristic mitochondrial enlargement and disarrayed cristae, despite severe cardiomyopathy. This may be attributed to the young age of the proband at the time of sample collection. Disease progression raised a clinical suspicion of Barth syndrome for which he was treated accordingly for 2 years. A manual re-inspection of the exome sequencing data revealed the short deletion in the non-coding 5'UTR of *TAFAZZIN*, confirming this diagnosis and highlighting the value of phenotype-driven genetic testing. The confirmation of Barth syndrome caused by a deletion in *TAFAZZIN* has enabled genetic screening to be offered to family members and the option of future reproductive options in female carriers.

Pathogenic variants leading to TAFAZZIN deficiency include nonsense, splice site, missense, and partial or full gene deletions. Partial deletions can offer insights into essential functional elements within a gene. In our proband, the deletion maintained transcriptional activity, indicating that the deleted region is not essential for RNA expression. Full-length TAFAZZIN is encoded by 11 exons, with multiple isoforms due to alternative splicing of exons 5 to 7, as well as two reported translational start sites, located in exons 1 and 3 (4). We did not detect truncated TAFAZZIN initiated from an alternative ATG start codon in our proband or in control hearts. Since the TAFAZZIN antibody epitope sequence is proprietary protected, we cannot rule out the possibility that a truncated protein exists to which the antibody does not bind. Previous studies in *Saccharomyces cerevisiae* showed that only full-length TAFAZZIN and an isoform lacking exon 5 retained catalytic activity and isoforms translated from the downstream AUG codon lacked functional activity (20). Therefore, deletion of the canonical start codon in our proband prevents the expression of catalytically active TAFAZZIN. An analysis of the breakpoint junction sequences revealed that the deletion likely occurred because of non-homologous end-joining in a GC-rich region with 2 base pair microhomology (Figure 1E). A similar mechanism reportedly explains a different deletion that encompasses the entire first exon of *TAFAZZIN*, NM_000116.3:c.-72_109+51del (19). Recombination between misaligned Alu elements is another common mediator of large partial and whole gene deletions in *TAFAZZIN.* Two such examples are a 13.7 kb whole *TAFAZZIN* gene deletion, with AluSx and AluSq repeat homologies found at the breakpoints in a person with severe cardiomyopathy and neutropenia (21), and a 9.9 kb deletion of the first five exons of *TAFAZZIN* at AluY and AluSx sequences in an individual with Barth syndrome, DCM, intermittent neutropenia, and lactic acidosis (19). There is no correlation between disease severity and *TAFAZZIN* variant class in Barth syndrome (2, 22).

This study is not without limitations. Control myocardial tissue for Western blot analysis was obtained from adult females, and we did not quantify the *TAFAZZIN* transcripts; therefore, the deletion may have impacted TAFAZZIN mRNA expression levels.

In summary, we report the case of a patient with Barth syndrome with progressive disease requiring heart transplantation due to a novel small deletion affecting the start codon of *TAFAZZIN*, providing further evidence of LoF variants in Barth syndrome. We demonstrated that the loss of the translation start site led to the loss of full-length TAFAZZIN without the initiation of translation from an in-frame downstream methionine. Our study emphasises the need for a careful analysis of coding and non-coding regions of *TAFAZZIN* during genetic testing on the suspicion of Barth syndrome.

## Data Availability

The datasets presented in this article are not readily available because it would not be in accordance with the ethical consent provided by the participant on the use of confidential and identifiable human genetic data and would compromise the anonymity of the participant given this is a case report of one individual. Requests to access the datasets should be directed to the corresponding author.
